# Testing the Impact of Phone Texting Reminders for Children's Immunization Appointments in Rural Cameroon: Protocol for a Nonrandomized Controlled Trial

**DOI:** 10.2196/47018

**Published:** 2023-08-09

**Authors:** Yayah Emerencia Ngah, Ghazal Raoufi, Maral Amirkhani, Ashkan Esmaeili, Rasa Nikooifard, Shidrokh Ghaemi Mood, Ava Rahmanian, Minyahil Tadesse Boltena, Eresso Aga, Ujjwal Neogi, George Ikomey Mondinde, Ziad El-Khatib

**Affiliations:** 1 Azire Integrated Health Center Bamenda Health District Ministry of Health Bamenda Cameroon; 2 Public Health Graduate Studies, Department of Biology The Bahá'í Institute for Higher Education Tehran Iran; 3 Armauer Hansen Research Institute Ministry of Health Addis Ababa Ethiopia; 4 UNICEF Afghanistan Kabul Afghanistan; 5 Department of Laboratory Medicine Karolinska Institutet Stockholm Sweden; 6 Center for the Studies and Control of Communicable Diseases Faculty of Medicine and Biological Sciences University of Yaoundé 1 Yaounde Cameroon; 7 Department of Global Public Health Karolinska Institutet Stockholm Sweden

**Keywords:** immunization, children, Cameroon, digital health, global health, nonrandomized controlled trial, child vaccination

## Abstract

**Background:**

Globally, over 20 million children are unvaccinated and over 25 million missed their follow-up doses during the COVID-19 pandemic; thus, they face vaccine-preventable diseases and unnecessary deaths. This is especially the case for those with HIV or living in vulnerable settings. Using cell phones to send reminders to parents has been shown to improve vaccination rates.

**Objective:**

We aim to determine whether implementation of an automated SMS reminder will improve child vaccination rates in a turbulent, semiurban/semirural setting in a low-income country.

**Methods:**

This will be a nonrandomized controlled trial that will be conducted at Azire Integrated Health Centre, Bamenda, Cameroon.

**Results:**

A total of 200 parents per study group (aged over 18 years) who are registered at the clinic at least one month prior to the study will be recruited. The intervention group will receive 2 reminders: 1 week and 2 days prior to the scheduled vaccination. For those who miss their appointments, a reminder will be sent 1 week after their missed appointment. The control group will receive the regular care provided at the clinic. Baseline information, clinical visit data, and vaccination records will be collected for both groups. Descriptive statistics will be used to summarize baseline characteristics between and within clusters and groups. The Fisher exact test will be used to compare parent-child units who return for follow-up visits (as a percentage) and children vaccinated as scheduled (as a percentage) between the study groups. Finally, we will compare how many members of both study groups return for 1 follow-up visit using Kaplan-Meier survival analysis.

**Conclusions:**

Due to limited effective child vaccination interventions in unstable settings, this study will be of high importance for suggesting a holistic approach to improve child vaccination and public health.

**International Registered Report Identifier (IRRID):**

DERR1-10.2196/47018

## Introduction

Child immunization is a key component of the World Health Organization (WHO) and United Nations Children’s Fund (UNICEF) child survival intervention package [1]. Immunizing children is an essential strategy in primary health care services and a crucial public health objective. Yet internationally, there are up to 19.4 million children who are still unvaccinated and face unnecessary deaths [2], including an especially high number of children living either in resource-limited settings or living with HIV. Therefore, children are considered a high-risk group for acquiring vaccine preventable diseases (VPDs) Also, the WHO and UNICEF warn of an increased number of missed follow-up doses for children due to the COVID-19 pandemic since 2020; over 25 million children have missed their follow-up doses. Such a decrease in child vaccination is considered a serious threat to the health of children, especially children living with HIV, in comparison to their immunocompetent similar-aged peers [3].

The vaccination system in Cameroon, including the administration of follow-up doses, has been weakened, and herd immunity has been compromised. In September 2022, the government of Cameroon announced a strategy to promote immunization among zero-dose children [4]. In 2021, UNICEF reported that different regions of Cameroon face the challenge of low vaccination coverage for several reasons, including armed conflict, insecurity, and underfunding of national vaccination programs [1]. Using cell phones to send reminders to parents about vaccination appointments has been shown to support adherence [5,6].

In 2019, we tested a smartphone app called Children Immunization App (CIMA) at a refugee camp in northern Jordan [6]. CIMA was developed to record the vaccination history of children according to the Jordanian national vaccination guidelines, provide health education information, and generate automated reminders for parents. The app was specially designed to target people with low literacy, with consideration of usability and technical concerns in a marginalized context [2]. CIMA was tailored to meet the needs of the target population. The messages and visuals were all customized to fit the local context [2].

In 2021, in collaboration with the University of Yaoundé and the United Nations Office on Drugs and Crime (UNODC) [7], we modified the CIMA functions. The penetration of smartphones is low in Cameroon; therefore, installing CIMA for parents would create a large selection bias. However, non-smartphone cell phones have an estimated penetration of over 95% [8]. Therefore, we have modified the app so information is saved locally in the clinic and reminders and health education messages are sent in the form of SMS texts to the registered cell phone numbers of the parents. We used English and French messages in order to fit the needs of the Cameroonian context. The main aim of this study is to implement an automated SMS system at Azire Integrated Health Centre, Bamenda. The objective is as follows: analyze the effect of the cell phone reminder intervention on the percentage of parent-child units returning for follow-up visits and the percentage of children receiving immunization as scheduled.

## Methods

### Study Setting

The study will be conducted at Azire Integrated Health Centre, Bamenda. Bamenda is located in the northwestern part of Cameroon (known for the volcanic Bamenda Highlands). Bamenda is considered a cosmopolitan town, with an area of 5250 km^2^; it includes 7 subdivisional councils and over 800,000 inhabitants. Since 2016, a sociopolitical crisis has been ongoing in Bamenda and it has become a turbulent zone. Today, it is estimated that over 95% of households in Bamenda own at least one cell phone.

The national vaccination schedule for children takes place during the first 11 months after birth at 5 clinic visits. The first contact is at birth and includes (1) the bacillus Calmette-Guérin (BCG) vaccine again tuberculosis and (2) the oral polio vaccine (OPV). The second contact is at 6 weeks and includes vaccination against diphtheria, tetanus, pertussis, hepatitis B, *Haemophilus influenzae* type b (DTP-HepB-Hib), OPV, pneumococcal infections (pneumococcal vaccine [PCV] 13 and rotavirus vaccine 1). The third contact is at 10 weeks and includes DTP-HepB-Hib, OPV, PCV 13, and rotavirus vaccine. The fourth contact is at 14 weeks and includes DTP-HepB-Hib, OPV, inactivated poliovirus vaccine, PCV 13, and rotavirus vaccine. The fifth contact is at 9 months and includes vaccination against measles, rubella, and yellow fever. Additionally, vitamin A is given during months 6 to 11 (Table 1) [9]. This study aims to assess the impact of an automated reminder at the second contact appointment.

The team members have different areas of expertise: epidemiology and the usefulness of digital tool reminders for clinic appointments for vaccination [3-5], the usefulness of telemedicine [6], adherence to taking antiretroviral treatment on time using cell phone reminders [7], and insulin supply pickup using SMS versus phone call reminders [8]; all members are experts in the study context of Bamenda and Cameroon.

**Table 1:** Vaccination schedule for children during the ages of 0 to 11 months in Cameroon.

Contact (age), vaccine			Preventable disease		Route of administration
**First contact (birth)**					
	Bacillus Calmette-Guérin vaccine	Tuberculosis		Intradermal	
	OPV^a^ 0	Poliomyelitis		Oral	
**Second contact (6 weeks)**					
	DTP-HepB-Hib^b^ 1	Diphtheria, tetanus, pertussis, infection due to *Haemophilus influenzae* type b, hepatitis B		Intramuscular	
	OPV 1	Poliomyelitis		Intramuscular	
	PCV^c^ 13 1	Pneumococcal infections		Oral	
	Rotavirus 1	Rotavirus diarrhea		Intramuscular	
**Third contact (10 weeks)**					
	DTP-HepB-Hib 2	Diphtheria, tetanus, pertussis, infection due to *Haemophilus influenzae* type b, hepatitis B		Intramuscular	
	OPV 2	Poliomyelitis		Intramuscular	
	PCV 13 2 ; rotavirus 2	Pneumococcal infections; rotavirus diarrhea		Intramuscular; oral	
**Fourth contact (14 weeks)**					
	DTP-HepB-Hib 3	Diphtheria, tetanus, pertussis, infection due to *Haemophilus influenzae* type b, hepatitis B		Intramuscular	
	OPV 3; IPV	Poliomyelitis		Oral; intramuscular	
	PCV 13 3	Pneumococcal infections		Intramuscular	
**Fifth contact (9 months)**					
	Measles, rubella	Measles, rubella		Subcutaneous	
	Yellow fever	Yellow fever		Subcutaneous	

^a^OPV: oral polio vaccine.

^b^DTP-HepB-Hib 1: diphtheria, tetanus, pertussis, hepatitis B, Haemophilus influenzae type b.

^c^PCV: pneumococcal vaccine.

### Sample Size

The current proportion of children returning back on time to complete their immunization in the clinic is estimated to be around 75%. We therefore aim to improve participation in the intervention clinic. With a study power of 80%, α=.05, and β=.2, we need to recruit a total of 400 participants (at a 1:1 ratio, ie, n=200 participants per study arm). We will include patients lost to follow-up in an intent-to-treat analysis strategy.

### Inclusion and Exclusion Criteria

All registered parent-child units who fulfill the following inclusion criteria will be invited to participate in the study: (1) registration at the clinic within the past month at the time of the study; (2) subsequent immunization scheduled for newborns; (3) parents aged more than 18 years; (4) at least one parent owns a cell phone, and (5) residency in the northwestern province. Potential participants will be excluded if the baby is coming for its second or later immunization visit or if the parents are not residents of the northwestern province.

### Intervention and Control Groups

The recruitment will be done through two steps. In step 1, the intervention group will be recruited. Then, in step 2, when the sample size is achieved, recruitment of the control group will be conducted until we reach the required sample size.

### Intervention Group

Study participants in the intervention group will receive the CIMA automated reminders on their cellphones. Messages will be sent to them to remind them of their appointments. Also, the CIMA system will provide educational information to the parents based on the parenting skills materials from the UNODC [7,10].

After obtaining informed consent, we will give each baby a study ID number that starts with the letter *I* (for “intervention”) and a continuous ID number that starts with the number 1001. Then, we will write the study ID number on a sticker that will be placed on the vaccination card of the newborn, so we can identify the baby in later visits as being enrolled in the CIMA intervention study group. For example, the first baby that will be enrolled in the intervention group will be given the ID number I-1001 and the last baby will have the ID number I-1201.

After the newborn receives the first vaccine, the vaccination nurse provides the parent an appointment date for the second contact vaccine. Then, we will record the appointment date in the CIMA database and schedule an automated reminder for the parents that will be sent at two time points: (1) 1 week before the appointment and (2) 2 days prior to the second contact appointment date. For example, if the second contact appointment date is scheduled for a Thursday, the automated SMS reminder will be sent out on the Thursday of the previous week and on the Tuesday of the same week. The content of the message will be as follows: “Dear parent, this is a kind reminder for the vaccination appointment of your child, scheduled on Thursday [date] at the Azire Integrated Health Centre.” This message has a total of 150 characters and can thus fit in 1 SMS; SMS messages have a maximum number of characters of 160. When the parents come to the second contact appointment, we will be able to identify enrolled newborns by the sticker that was placed on the vaccination card. We will record the date of arrival at the second contact appointment in the CIMA database. Each week, we will auto-generate a list of newborns that have missed their second contact appointment, and we will send a reminder to the parents about missing the appointment. The content of the message will be as follows: “Dear parent, your child has missed the vaccine appointment this week. You are requested to come as soon as possible to the Azire Integrated Health Centre”; this message includes a total of 153 characters and can fit into 1 SMS.

Each week, the parent will receive one SMS that has an educational message on parenting skills. We will use the UNODC parenting skills toolkit [9], which was tested in the past with CIMA in Jordan [10]. The last SMS educational message will be sent out on week 4 of the study (Table 2).

**Table 2:** Overview of the action plan for the intervention and control groups.

			Study week													
			0^a^		1	2		3		4		5		6^b^		7
**Intervention group**																
	Explain the study, ask for consent, administer the questionnaire (Multimedia Appendix 1)	✓														
	Register the cell phone number of the parent in the CIMA^c^ database	✓														
	SMS education message			✓		✓	✓		✓							
	SMS reminder for the appointment										✓					
	Automatically scheduled reminder 1 week prior the appointment												✓			
	Mark in the CIMA registry if the newborn came back on time												✓			
	SMS reminder for parents that have missed the appointment														✓	
**Control group**																
	Explain the study, ask for consent, administer the questionnaire (Multimedia Appendix 1)	✓														
	Register the expected date for the second contact (for comparison with the intervention group)	✓														
	Mark the date when the newborn came back to the clinic												✓			

^a^First contact.

^b^Second contact.

^c^CIMA: Children Immunization App.

### Control Group

Participants in the control group will continue to receive regular routine messages from the health facility. After obtaining consent, we will use the same procedure to give ID numbers to the babies in the control group, except that the letter *C* (for “control”) will be used instead of *I*. The control group will receive usual care at the clinic. For example, the vaccination nurse will review the schedule of vaccine appointments at the end of the month, then call parents that have missed their baby’s appointment to remind them about the second contact vaccine appointment and to invite them to come back to the clinic. No educational messages and no SMS reminders will be sent out to the participants enrolled in the control group.

### Measures

The key measures for this study are the percentage improvement in parent-child units who return for follow-up visits and the percentage improvement in children who receive immunization as scheduled. We will send reminders to the parents of children that do not come back to their appointments within 1 week after the scheduled appointment.

### Data Collection

At the time of study enrollment, we will approach parents of newborns coming for their first immunization dose. In the intervention and control arms, we will collect baseline information using a questionnaire (Multimedia Appendix 1). As we plan to monitor adherence to vaccination appointments from birth to the age of 6 months, we will use the clinic ID number to monitor visits using the clinic health care records for both study groups.

Both clinical visits and vaccination attendance will be collected from the health facility registration records. Informed consent will be obtained at the baseline visit from clients after an explanation of the study purpose and the data analysis. No personal identifiers will be collected (ie, names or personal addresses), but we will collect the phone numbers of one of the parents, as this will be used to send the SMS messages. All data will be stored in a secured location. No financial compensation will be offered to the parents, and they will have the choice to ask to stop being enrolled in the study.

### Data Collection Tool

Data will be collected from the clinic registration books for the upcoming second contact appointment visit. The baseline questionnaire (Multimedia Appendix 1) will be used to collect basic demographic and household information, including the type of household (rented house, rented apartment, informal settlement, other, or no answer), date of birth of the father and mother, level of education of the father and mother (don’t know how to read and write; know how to read and write; primary or elementary school; secondary school; postschool technical level; university level; don’t know or don’t remember, or refused to answer), the availability of a private toilet inside the household (yes or no); professional status of the father and mother (full-time job, part-time or hourly job, self-employed, do not work, returned, or refused to answer), total number of children living at home, who is the decision-maker for vaccination (both parents, mother, father, or other); total number of children younger than 5 years at home; and amount of money spent by the household in the month prior the date of the interview.

### Data Analysis

The data analysis will be conducted in 3 steps. First, we will summarize the characteristics of the babies and their parents using a general descriptive statistical analysis and compare these characteristics between the intervention and control groups. We will use the Fisher exact test to detect any statistically significant differences in the key measures between the intervention and control groups (*P* values <.05 will be considered statistically significant). Second, we will calculate the attrition rate of the babies and compare the attrition rate in each of the intervention and control groups. Third, we will compare how often both study groups return for 1 follow-up visit using a Kaplan-Meier survival analysis. We will determine the proportion of babies that did come back on time at the end of each week to help the clinic to visually identify the proportion of latecomers and which geographical areas they live in. We will use R (R Core Team) for the statistical analysis. For the descriptive analysis, we will use the *dplyr* library package in R; for the Fisher exact test, we will use the *stats* library package; for the Kaplan-Meier survival analysis, we will use the *survival* library package.

### Ethical Considerations

Ethical approval was received from the Cameroon Ministry of Public Health, Northwest Regional Delegation of Health (324/ATT/NWR/RDPH/BRIGAD).

## Results

Study enrollment was planned for the beginning of 2023. Usually, the clinic schedules child vaccination 2 days per week (Tuesdays and Thursdays). Approximately 20 of 50 babies are newborns and receive their first-contact vaccine (Table 1). This means that we estimate that we will need to approach 40 of 100 parents per week to explain the study and ask for consent. We estimate we will recruit a total of 200 babies in each study group during a total period of 5 weeks (pending the response rate per week of the parents). Therefore, the total duration of recruitment might be between 10 and 12 weeks.

**Figure 1:**
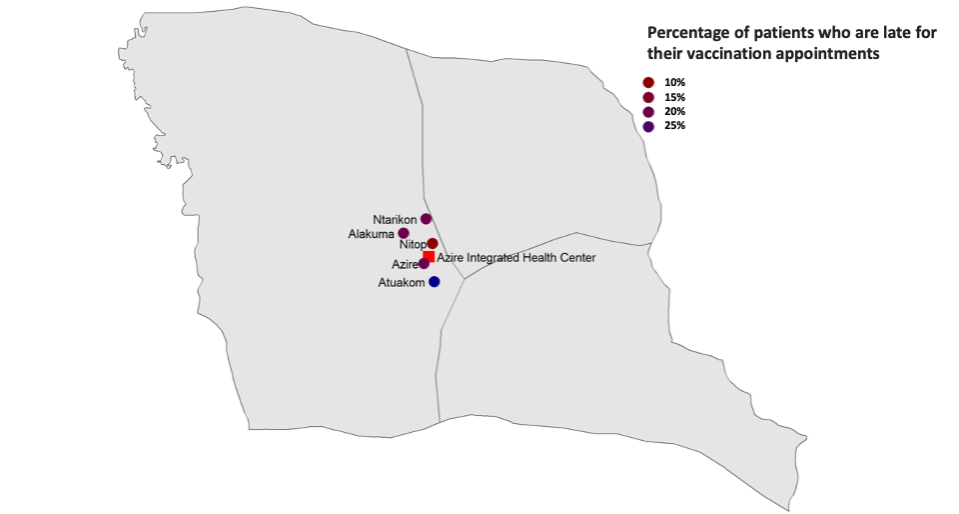
Fictional figure projecting how the data for patients in Bamenda who are late for their vaccination appointments will be visualized.

We expect to complete the study by July 2023 and complete the data analysis and submission of the final report by 2024. We include a dummy example of how the data will be visualized for the vaccination clinic site (Figure 1). The general characteristics of the babies in both study groups are presented in a table, including the characteristics of the babies that did not come back to the clinic. The Kaplan-Meier survival analysis will be shown as a figure.

## Discussion

### Anticipated Findings

Child immunization is a crucial public health objective and an essential strategy in primary health care services. However, globally, there are still millions of children who are unvaccinated and at risk for VPDs. Prior to the COVID-19 pandemic, the effectiveness of automated reminders for children attending vaccination appointments was tested either by the team members of this project in the context of a refugee camp in Jordan [6,7] and the general community in Canada [3] or by other authors in Ethiopia [11], Nigeria [12], and other contexts [13,14].

The COVID-19 pandemic has also led to an increase in the number of missed follow-up doses for children. In Cameroon, the vaccination system has been weakened, and herd immunity is compromised. Also, UNICEF has warned about the impact of spreading false information (known as misinformation) about national vaccination programs in Cameroon [15]. The spreading of misinformation is considered one of the major threats to public health interventions, especially during and after COVID-19.

In this paper, we described version 1.0 of a study protocol for a nonrandomized controlled trial of child vaccination reminders in a turbulent, semiurban/semirural setting in a low-income country. The main goal of the study is to implement an automated SMS reminder system at the Bamenda primary health care center. The objectives of the study are to analyze the effect of the cellphone reminder intervention on the percentage of parent-child units returning for follow-up visits and the percentage of children receiving immunization as scheduled. Instability in northwestern Cameroon makes it important to improve childhood vaccination, and to the best of our knowledge, this is the first study to support a national vaccination program by (1) using an automated system to send reminders in the form of SMS messages and (2) send health educational messages about vaccines, as well as about parenting skills. Ames et al [16] found that childhood vaccination communications have focused on national campaigns against polio, instead of routine vaccination programs. Therefore, we think this study might have the secondary impact of promoting routine vaccination among parents.

We have previously tested this concept among refugees at the Zaatari camp in Jordan, a vulnerable group [5]. We used the same questionnaire in Jordan but provided CIMA in the form of an Android smartphone app because the majority of households in the camp owned at least one Android smartphone. The market share of smartphones in sub-Saharan Africa is growing, but they have not yet reached the majority of the population; therefore, we are using an SMS reminder system. The authors have experience in the field setting up such systems in vulnerable contexts, such as in the Central African Republic [17].

The adoption of digital health solutions is becoming important and a crucial means of enhancing the health care system in general. The testing of digital health for the improvement of childhood vaccination programs in resource-limited settings will be a great help in improving equity of access to vaccination [18]. So far, digitization has not been done in a systematic fashion, and this study in Cameroon will contribute to the learning process on how to implement and use such digital health solutions in resource-limited settings.

The outcome of this study will be of high importance for finding a holistic approach to the improvement of childhood vaccination schedules in unstable contexts such as northwest Cameroon. Parents experience stress, especially during times of uncertainty. Therefore, this study will support new studies on assessing perceptions among parents of parenting-skills health education messages sent in the form of regular SMS messages. These messages were developed by the UNODC and have been tested in different contexts around the world, including in our study of CIMA in the Zaatari camp in Jordan [5]. In our planned study in Cameroon, we will send the messages via SMS, which will be done for the first time. Finally, the results of this testing may lead to the development of a holistic approach and eventually a tool to digitally register, enhance, and monitor the vaccination schedule of children in settings such as Cameroon.

We should note that the outcome of this study will open new opportunities to explore the usefulness of automated SMS reminder systems in resource-limited settings. Childhood vaccination programs have been weakened during the COVID-19 pandemic, and we hope that our intervention will empower both parents and the vaccination clinic staff to improve vaccination coverage [19]. Finally, by improving vaccination coverage, we will be able to reduce the risk of VPD-related outbreaks in resource-limited settings.

### Limitations

We foresee the following points as potential limitations of our planned study: (1) The language context of the study is predominantly English, so the study may not be generalizable to the rest of Cameroon, where French is spoken. (2) We plan to test the effect of the SMS reminders for 1 vaccination appointment only, so further studies will be needed to ensure effectiveness over longer periods of time (for example, to ensure that parents will not have SMS fatigue). (3) The scalability of the SMS reminders needs to be evaluated from a cost-effectiveness perspective to ensure that we can provide an estimated cost for policymakers if the Ministry of Health of Cameroon scales the project (however, we performed a cost-effectiveness analysis for the CIMA-Jordan project and found that the cost was low in the context of a low-income country [20]). (4) Bamenda is considered a turbulent location due to the current crisis; this might affect the study if the situation becomes more turbulent. Such situations, labeled “armed conflicts,” are known strongly impact childhood vaccination [21], as they may lead to either forced migration of the parents out of Bamenda or short-term closure of the Azire Integrated Health Centre.

### Conclusion

There is limited evidence on effective interventions to improve childhood vaccinations in turbulent settings [14]. It is crucial to ensure parents receive reminders and correct information about all vaccines. Therefore, this project will have an impact on the health of the children and on the public health system. Also, we expect that this study will support the work of sharing correct information with parents. After the end of this study, we foresee planning to expand the implementation of CIMA to other primary health care centers in Cameroon in order to reach a larger population of children, including an evaluation of the effectiveness of the SMS reminder intervention on vaccination coverage and adherence to follow-up visits. Additionally, we will explore the potential for integrating CIMA with other health care systems, such as electronic medical records, to improve data collection and analysis.

## Appendix

Multimedia Appendix 1 Questionnaire for baseline information for the Children Immunization App (CIMA).

## Abbreviations

BCG: bacillus Calmette-Guérin
CIMA: Children Immunization App
DTP-HepB-Hib: diphtheria, tetanus, pertussis, hepatitis B, Haemophilus influenzae type b
OPV: oral polio vaccine
PCV: pneumococcal vaccine
UNICEF: United Nations Children’s Fund
UNODC: United Nations Office on Drugs and Crime
VPD: vaccine-preventable disease
WHO: World Health Organization

## Data Availability

The data sets generated during and/or analyzed during this study are available from the corresponding author on reasonable request.
