# Supplementary material for: Testing the Impact of Phone Texting Reminders for Children's Immunization Appointments in Rural Cameroon: Protocol for a Nonrandomized Controlled Trial
Source: JMIR Res Protoc. 2023 Aug 9;12:e47018. doi: 10.2196/47018 (PMC10448290; doi:10.2196/47018)
Supplement: Multimedia Appendix 1 [file resprot_v12i1e47018_app1.docx]

**Multimedia Appendix 1 – Questionnaire for baseline information for the Children Immunization App (CIMA)**

Date of survey (DD/MM/YYY): _____ / _____ / __________

Study ID: __________

What phone number should we use to send you SMS reminders and health education messages?

____________________________________________

**Name of your district**:

**Demographics of the parents**

1. Do you live in:

( ) a. Rented house ( ) b. Rented apartment

( ) c. Informal settlement ( ) d. Other ____________________

( ) e. Did not answer

2. Do you have a private toilet inside your household?

( ) Yes ( ) No

3. Information about mothers and fathers

|  | **Mother** | **Father** |
| --- | --- | --- |
| **Date of birth** | __________ dd/ mm/ yyyy | __________ dd/ mm/ yyyy |
| **Education level** | ( ) Don’t know how to read and write  ( ) Knows how to read and write  ( ) Primary/elementary school  ( ) Secondary school  ( ) Post school technical level  ( ) University level  ( ) Don’t know/don’t remember  ( ) Refused to answer | ( ) Don’t know how to read and write  ( ) Knows how to read and write  ( ) Primary/elementary school  ( ) Secondary school  ( ) Post school technical level  ( ) University level  ( ) Don’t know/don’t remember  ( ) Refused to answer |
| **Professional status** | ( ) Full-time job  ( ) Part-time/hourly job  ( ) Self-employed  ( ) Do not work  ( ) Retired  ( ) Refused to answer | ( ) Full-time job  ( ) Part-time/hourly job  ( ) Self-employed  ( ) Do not work  ( ) Retired  ( ) Refused to answer |

**4.** **Who is the head of the household?**

( ) Husband ( ) Wife

5. Who in your family makes decision about vaccinating your child? (one answer only)

( ) Both parents ( ) Mother ( ) Father

( ) Other, please specify _______________

**5.** **What is your total number of children under 5 years of age at home?**____

**6. How much money did you spend last month on your household? ______**
